# Beyond histocompatibility: human leukocyte antigen as a cross-disciplinary platform for immune recognition and clinical decision-making

**DOI:** 10.3389/fimmu.2026.1931212

**Published:** 2026-09-08

**Authors:** Saikat Mandal, Joyisa Deb, Aswin K. Mohan, Suhasini Sil, Manideepa Maji, Shirin Hasan, Fahad Hassan Ashraf, Arkadeep Dhali

**Affiliations:** 1 Translational Medical Sciences, School of Medicine, University of Nottingham, Nottingham, United Kingdom; 2 NIHR Nottingham Biomedical Research Centre, Nottingham University Hospitals NHS Trust, Nottingham, United Kingdom; 3 Transfusion Medicine & Blood Centre, Apollo Excelcare Hospital, Guwahati, India; 4 Transfusion Medicine, All India Institute of Medical Sciences, Bibinagar, India; 5 Transfusion Medicine, Rajiv Gandhi Super Speciality Hospital, New Delhi, India; 6 Hull York Medical School, University of Hull, Hull, United Kingdom; 7 Haematology, Hull University Teaching Hospitals NHS Trust, Hull, United Kingdom; 8 Queen’s Centre for Oncology, Hull University Teaching Hospitals NHS Trust, Hull, United Kingdom; 9 HPB Medicine and Gastroenterology, Nottingham University Hospitals NHS Trust, Nottingham, United Kingdom; 10 Division of Cancer, Imperial College London, London, United Kingdom; 11 Academic Gastroenterology, Sheffield Teaching Hospitals NHS Trust, Sheffield, United Kingdom

**Keywords:** cancer immunology, donor-specific antibodies, eplet matching, haematopoietic stem cell transplantation, HLA, HLA evolutionary divergence, HLA-E, HLA-G

## Abstract

Human leukocyte antigen (HLA) molecules mediate peptide presentation and shape immune recognition, with clinical relevance well beyond donor-recipient matching. This review describes how HLA variation and anti-HLA immunity affect transfusion medicine, autoimmune and infectious diseases, pharmacogenomics, cancer, pregnancy, solid organ transplantation and haematopoietic stem cell transplantation (HSCT). It outlines HLA nomenclature and testing, including sequence-based typing, single-antigen bead assays, virtual crossmatching and molecular mismatch analysis, and considers how population-specific allele and haplotype frequencies affect interpretation. An HLA result must be interpreted in clinical context, including exposure history, donor specificity, antibody strength, assay characteristics, organ type, molecular mismatch, HLA evolutionary divergence, immunosuppression and ancestry. In transfusion medicine, recipient alloimmunisation can cause platelet refractoriness and complicate future transplantation, whereas donor antibodies contribute to transfusion-related acute lung injury. Transfusion-associated graft-versus-host disease is a different cellular complication. In autoimmunity, type 1 diabetes and systemic lupus erythematosus show how HLA class II variation can influence peptide selection, immune tolerance and autoantibody profiles, although most associations are not diagnostic alone. Pharmacogenomic testing has clearer clinical value for selected drugs, including abacavir, carbamazepine and allopurinol. In cancer, loss of classical HLA expression and non-classical HLA-E and HLA-G pathways contribute to immune escape and are being explored as therapeutic targets. In transplantation, HLA assessment increasingly combines donor-specific antibodies, crossmatch results, molecular mismatch and organ-specific risk. Eplet, amino-acid and predicted indirect T-cell epitope mismatch can support risk assessment at population level, but no single score should determine organ allocation or immunosuppression. HSCT differs because the graft transfers a donor immune system, creating competing risks of graft failure, graft-versus-host disease and relapse. Clinical implementation requires reliable assays, validated clinical associations, evidence of patient benefit, multi-ancestry validation, consistent reporting and equitable access. We distinguish established clinical practice from developing risk-stratification methods and early mechanistic findings.

## 1 Introduction

The human leukocyte antigen (HLA) gene complex lies within the major histocompatibility complex (MHC) on chromosome 6p21.3 and encodes the principal human molecules responsible for peptide presentation and immune recognition. It includes classical class I genes (HLA-A, HLA-B, and HLA-C), classical class II genes (including HLA-DR, HLA-DQ, and HLA-DP), non-classical HLA genes, and immune-related genes within the broader MHC region (1–3).

**Figure 1:**
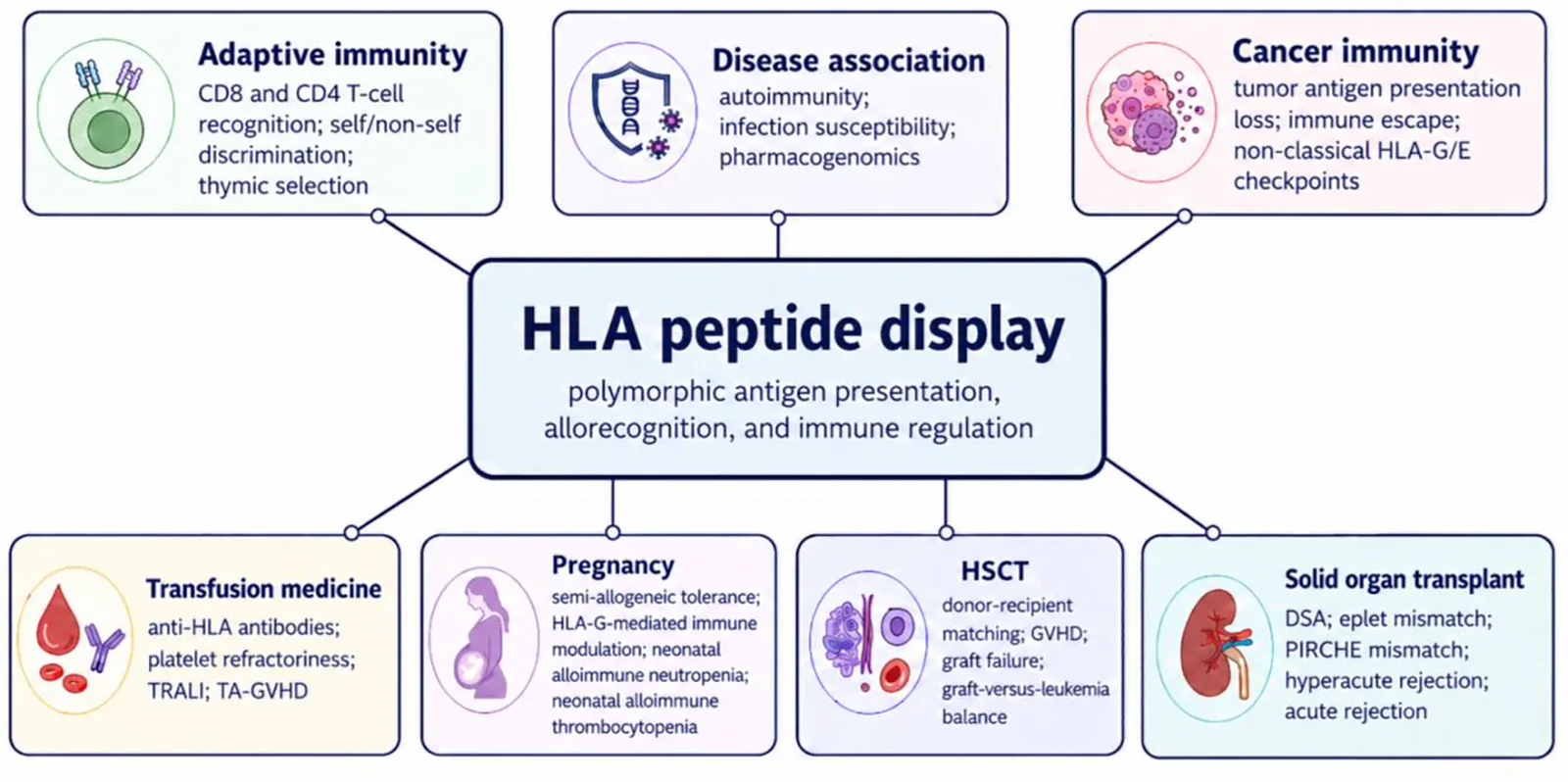
Conceptual framework for HLA as a bridge across clinical immunology. HLA peptide display and immune regulation link adaptive immunity to transfusion alloimmunisation, disease susceptibility, pharmacogenomics, cancer immune escape, pregnancy, solid organ transplantation and HSCT.

The clinical relevance of HLA reflects its extreme polymorphism, its expression on immune-relevant cellular targets, and its direct role in antigen-specific recognition. A single allele can influence antiviral peptide presentation, autoimmune risk, drug hypersensitivity, platelet transfusion response, tumour visibility to cytotoxic T-lymphocytes and recognition of transplanted tissue as foreign (4). The broader clinical applications of HLA across different disciplines are summarised in **Figure 1**.

This review examines how HLA biology is applied across clinical disciplines. It considers how allele-level variation, anti-HLA antibodies, molecular mismatch, HLA evolutionary divergence and non-classical HLA-mediated regulation inform decisions in transfusion practice, disease association, pregnancy, cancer immunity, solid organ transplantation and haematopoietic stem cell transplantation.

## 2 Narrative review approach and scope

This narrative review links HLA biology with clinical decision-making across transfusion medicine, autoimmune and infectious disease, pharmacogenomics, cancer immunology, pregnancy, solid organ transplantation and haematopoietic stem cell transplantation. It is organised around five questions: how HLA shapes immune recognition; how HLA typing and antibody testing should be interpreted; how alloimmunisation affects transfusion support; how HLA variation influences disease susceptibility, drug hypersensitivity and cancer immune surveillance; and how HLA information guides organ and cellular transplantation. Foundational studies, nomenclature and laboratory guidance, major reviews, consensus statements and clinically relevant contemporary studies were prioritised.

Interpretation of HLA associations requires caution because the MHC is highly polymorphic, ancestry-structured and characterised by extensive linkage disequilibrium. An association was considered clinically actionable when it was supported by replicated evidence, a plausible mechanism and a defined effect on clinical decision-making. Some HLA findings have entered routine prescribing, transfusion or transplant pathways; many disease associations and molecular scores remain most useful for mechanistic interpretation or research.

### 2.1 Approach to evidence appraisal

As application of HLA-related testing ranges from routine clinical testing to early mechanistic discovery, the evidence is considered according to its readiness for clinical practice. Applications are regarded as established when findings have been reproduced, testing is standardised or supported by guidelines, and the result leads to a clear change in clinical management. Evolving applications have a credible biological basis and reasonably consistent prognostic associations, but still lack agreed thresholds, prospective implementation studies or evidence that test-guided care improves patient outcomes. Exploratory applications are supported mainly by mechanistic studies, small cohorts, single-centre analyses or early translational work and are therefore discussed as priorities for further research rather than as tools ready for routine use.

For each domain, emphasis is placed on study design, independent replication across populations, reproducibility, assay standardisation and demonstrable clinical utility. This framework is used throughout the review and is summarised at the end of the review, so that association, prognostic value and clinical actionability are not treated as equivalent.

## 3 Genomic architecture, polymorphism and nomenclature

The HLA region is among the most gene-dense and polymorphic regions of the human genome. Class I molecules are expressed on nearly all nucleated cells and present endogenous peptides to CD8 T cells. Class II molecules are expressed mainly by antigen-presenting cells and present exogenous or vesicular peptides to CD4 T-cells. Non-classical class I molecules such as HLA-E, HLA-F and HLA-G are less polymorphic and often act as regulatory ligands for natural killer (NK) cells and other immune subsets. The class III region does not encode peptide-presenting HLA molecules but contains complement and inflammatory genes, underscoring the broader immunological significance of the MHC locus (1, 4).

According to the Immuno Polymorphism Database-International ImMunoGeneTics/HLA Database (IPD-IMGT/HLA), more than 43,000 unique HLA alleles from 47 genes are now recognised by the World Health Organisation Nomenclature Committee for Factors of the HLA System, and the database continues to grow through curated submissions and standardised nomenclature (2, 3). Extensive HLA polymorphism is considered an evolutionary adaptation to maximise population-level immune protection against pathogens. This diversity also complicates clinical practice by increasing the complexity of laboratory typing, donor matching, disease-association analysis, and equitable donor selection.

### 3.1 Population genetics and HLA distribution

HLA allele and haplotype frequencies vary markedly among and within populations as a consequence of balancing selection, demographic history, founder effects, migration, admixture and recombination. These differences are clinically relevant. In a registry analysis of 130,518 Indian stem-cell donors, eight linguistic-geographical groups formed distinct southern, north-western and Bengali clusters, with corresponding differences in donor-match probabilities. High-resolution class I data from eastern and southern Africa likewise demonstrated substantial between-country and within-country diversity and showed that African-American reference data cannot be assumed to represent African populations (5, 6).

Population structure can bias HLA-disease associations, pharmacogenomic effect estimates, imputation accuracy, vaccine and neoantigen prediction, and estimates of donor availability. It also influences calculated panel-reactive antibody, unacceptable-antigen frequencies and the equity of registry-based matching or organ allocation. Studies should therefore report typing resolution, recruitment population and ancestry methods; control for population stratification and relatedness; and validate associations, risk scores and molecular-mismatch thresholds in independent, ancestrally diverse cohorts. Broad racial or ethnic categories should not be treated as interchangeable with genetically informed population structure.

### 3.2 HLA nomenclature and typing resolution

**Figure 2:**
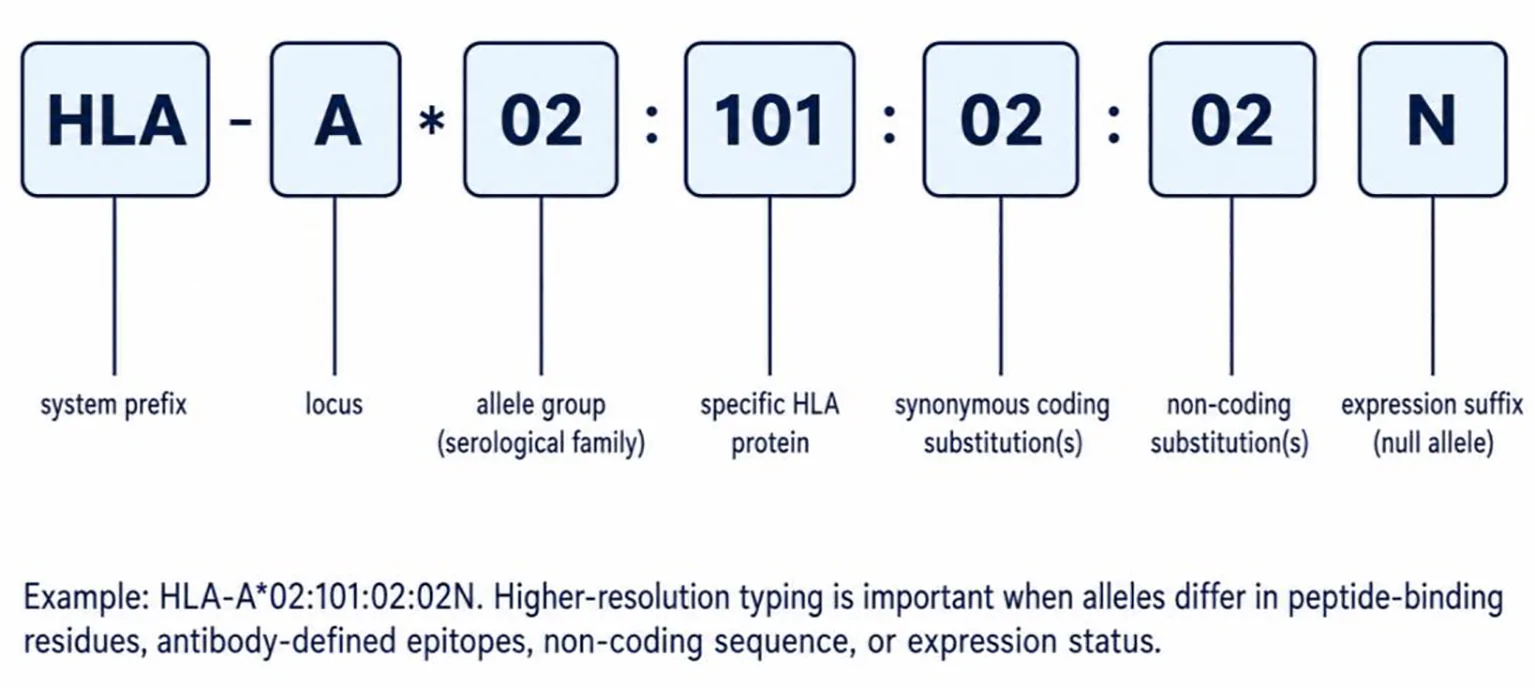
Figure 2 illustrating the structure of HLA allele nomenclature using the example HLA-A*02:101:02:02N. The diagram labels the system prefix, locus, allele group, specific HLA protein sequence, synonymous coding variation, non-coding variation, and expression suffix. The fields are arranged sequentially to show increasing molecular details.

Modern HLA nomenclature encodes increasing levels of molecular precision. In a name such as HLA-A*02:101:02:02N, the locus appears before the asterisk; the first field denotes the allele group; the second field defines a unique protein sequence; the third field captures synonymous coding variation; the fourth field captures non-coding variation; and the suffix provides expression information, such as N for a null allele (**Figure 2**). The level or resolution of typing required depends on the clinical context. Identification of a single high-risk allele may be sufficient for pharmacogenomic screening, whereas unrelated haematopoietic stem cell transplantation (HSCT) and molecular mismatch analysis usually require high-resolution or extended typing (2, 7, 8).

## 4 HLA as an immune recognition system

HLA molecules are central to immune surveillance because they determine which peptides are made visible to the immune system. For HLA class I, intracellular proteins are degraded by the proteasome, transported into the endoplasmic reticulum by the transporter associated with antigen processing (TAP), loaded onto class I molecules in the presence of β-2-microglobulin, and displayed at the cell surface. These complexes are surveyed mainly by CD8-positive T-cells. For HLA class II, exogenous or vesicular proteins are processed within endosomal compartments and loaded onto class II molecules after invariant-chain removal and HLA-DM-mediated peptide editing. These complexes are recognised mainly by CD4-positive T-cells and support T-cell helper response, antibody maturation, macrophage activation and immune memory (1, 9). The major HLA classes and their principal clinical/biological implications are summarised in **Table 1**.

**Table 1:** HLA classes and selected clinical implications.

Region/class	Main molecules	Core biological role	Selected clinical relevance
Class I	HLA-A, -B, -C	Endogenous peptide presentation to CD8 T cells; ligands for selected NK-cell receptors	Viral control, cancer immune surveillance, solid organ and HSCT matching, platelet refractoriness
Non-classical class I	HLA-E, -F, -G	Immune regulation, NK/T-cell inhibition, maternal-fetal tolerance	Pregnancy tolerance, cancer immune escape, HLA-E/NKG2A and HLA-G/immunoglobulin-like transcript checkpoint biology, emerging transplant-tolerance strategies
Class II	HLA-DR, -DQ, -DP	Exogenous/vesicular peptide presentation to CD4 T cells	Autoimmune disease association, antibody formation, HLA-DQ donor-specific antibody (DSA), HSCT donor selection
Class III region	Complement and inflammatory genes	Inflammation and immune effector pathways rather than peptide presentation	Disease association through linkage and immune regulation

The HLA system also shapes the T-cell repertoire before immune challenge. During thymic selection, developing T-cells survive only if their receptors recognise self-HLA with appropriate affinity. Clones with no useful HLA interaction are neglected, whereas clones with excessive self-reactivity are deleted or diverted into regulatory lineages. Many HLA-disease associations can be understood through this framework: an allele may present microbial peptides efficiently, but may also present self, altered-self or drug-modified peptide repertoires in a way that favours autoimmunity or hypersensitivity (4, 9).

HLA-mediated recognition extends beyond conventional T-cell responses. Classical HLA-C and selected HLA-A and HLA-B molecules also act as ligands for killer cell immunoglobulin-like receptors (KIRs), allowing natural killer (NK) cells to sense changes in HLA class I expression and contribute to the recognition of infected, stressed or transformed cells while preserving self-tolerance. Non-classical HLA molecules provide an additional layer of immune regulation, with their disease-specific roles discussed in later sections. Clinically relevant consequences include tumour immune escape through loss or downregulation of HLA class I and maternal-fetal tolerance mediated by HLA-G (10, 11). HLA therefore functions as both a peptide-presentation system and a broader regulator of adaptive and innate immune recognition.

### 4.1 HLA evolutionary divergence as a cross-cutting metric

HLA evolutionary divergence (HED) extends the conventional distinction between HLA homozygosity and heterozygosity by quantifying the amino-acid distance between the two alleles at a locus, commonly using Grantham distance. It is used as a proxy for the breadth and biochemical diversity of the immunopeptidome: two heterozygous alleles may be closely related or markedly divergent, with potentially different consequences for peptide presentation (12–15).

The clinical implications of HED depend on context. Higher class I HED has been associated with broader neoantigen presentation and improved immune checkpoint inhibitor outcomes, whereas donor class I HED has been linked to liver allograft rejection. In HSCT, HED may influence relapse, graft-versus-leukaemia effects and graft-versus-host disease, while HLA-DP diversity has been associated with vaccine responsiveness after transplantation (12–15). These observations are derived mainly from retrospective cohorts that differ in the loci, endpoints and treatment platforms examined. No prospective study has shown that an HED-guided decision improves patient outcomes; its use should therefore remain investigational until organ-, disease- and platform-specific thresholds are validated.

## 5 HLA testing: from serology to molecular and functional interpretation

HLA testing has evolved from serological antigen assignment to high-resolution molecular typing. Although serology remains useful in selected urgent or resource-limited settings, it cannot reliably distinguish alleles that differ at peptide-binding residues. Polymerase chain reaction-based methods, Sanger sequencing and next-generation sequencing (NGS) provide increasing resolution; NGS is now particularly relevant to transplant registries, pharmacogenomics and molecular mismatch analysis (7, 8, 16). The principal HLA testing modalities and their limitations are summarized in **Table 2**.

**Table 2:** Practical HLA testing modalities and interpretive cautions.

Testing domain	Examples	What it answers	Interpretive caution
HLA Typing	Serology, polymerase chain reaction with sequence-specific primers (PCR-SSP), sequence-specific oligonucleotide probes (SSOP), Sanger sequencing, NGS	Which HLA antigens/alleles are present?	Typing resolution must match the clinical question; low-resolution typing may miss clinically relevant protein differences.
Cell-based antibody testing	CDC-XM, FC-XM	Does recipient serum bind donor cells and/or fix complement?	CDC-XM is highly specific but relatively insensitive; FC-XM is more sensitive but may yield false-positive results because of non-HLA antibodies or cell/assay factors.
Solid-phase antibody testing	Luminex panel-reactive antibody (L-PRA), L-SAB	Which antibody specificities are present and are they donor-specific?	MFI is semi-quantitative; prozone/complement interference, denatured antigen and epitope sharing may affect results.
Integrated compatibility	Virtual crossmatch, unacceptable antigens, calculated panel-reactive antibody (cPRA)	Is a proposed donor immunologically acceptable?	Requires donor HLA typing, recent recipient antibody testing, exposure history, and clinical context.
Molecular mismatch	Eplet analysis; Predicted Indirectly Recognisable HLA Epitopes (PIRCHE-II) algorithm for HLA class II molecules	What is the predicted immunological disparity between donor and recipient HLA?	Useful for risk stratification, but clinically actionable thresholds require organ & population-specific validation.

Antibody testing addresses whether anti-HLA antibodies are present, whether they are donor-specific and whether they are likely to be clinically injurious. Complement-dependent cytotoxicity crossmatch (CDC-XM) detects cytotoxic complement-fixing antibodies with high specificity but limited sensitivity. Flow cytometric crossmatch (FC-XM) is more sensitive but may detect non-HLA or assay-related binding. Luminex single-antigen bead (L-SAB) assays define antibody specificity, but mean fluorescence intensity (MFI) is semi-quantitative and is influenced by epitope sharing, complement interference, prozone, bead saturation, immunoglobulin subclass and laboratory method (17–19).

**Figure 3:**
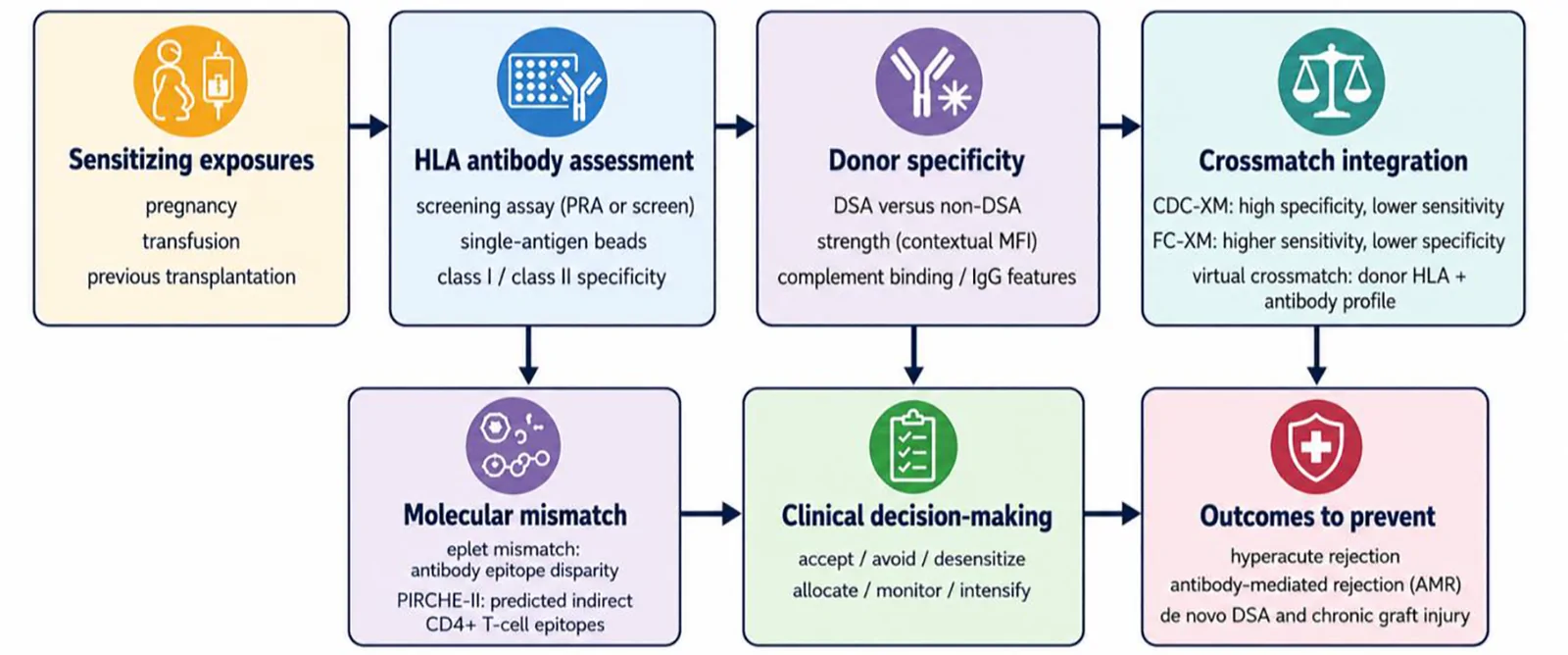
HLA-directed transplant immunology from sensitising exposure to clinical decision. The figure emphasises that donor-specific antibody assessment, crossmatch interpretation, and molecular mismatch analysis should be integrated with organ type, urgency and clinical context.

A 350-case renal transplant assay-comparison study demonstrates the complementary information provided by different platforms (20). T-cell FC-XM showed sensitivity and specificity of 71.43% and 91.50% for class I L-SAB positivity, whereas T-cell CDC-XM showed 32.14% and 98.64%; B-cell FC-XM and CDC-XM showed 94.94%/61.99% and 44.30%/94.83%, respectively. A 1,007-crossmatch deceased-donor analysis likewise found poor CDC-XM sensitivity for several donor-specific antibody patterns and substantial false-positive reactivity, while a living-donor cohort showed that MFI thresholds correlated with physical crossmatch positivity but did not behave as universal cut-offs. These findings support parallel interpretation of cell-based and solid-phase assays, with thresholds treated as laboratory- and context-specific decision-support values (20–22). These reports are retrospective, predominantly single-centre assay-comparison studies. They define relative analytical performance and local operating characteristics, but they do not establish universal thresholds or demonstrate that one testing strategy improves graft outcomes. An integrated approach to HLA-directed transplant immunology, incorporating sensitising exposures, antibody assessment, crossmatching and molecular mismatch, is summarised in **Figure 3**.

## 6 HLA in transfusion medicine

### 6.1 Transfusion as an HLA-sensitising exposure

Blood transfusion is an important source of HLA sensitisation because recipients are exposed to donor leukocytes, platelets and residual HLA-bearing cellular material. Red cells express little clinically relevant HLA, but red-cell concentrates may contain residual leukocytes, and platelet components retain abundant HLA class I. Leukoreduction reduces leukocyte exposure but does not eliminate platelet HLA exposure or completely prevent alloimmunisation.

The clinical consequences of transfusion-related HLA alloimmunisation are best established in chronically transfused patients, those with haematological malignancy or marrow failure, individuals with haemoglobinopathies and transplant candidates. Recipient anti-HLA antibodies can cause platelet refractoriness, restrict compatible platelet availability and complicate future transplantation by increasing calculated panel-reactive antibody (cPRA), creating unacceptable antigens and generating donor-specific antibody (DSA) risk. Donor anti-HLA or anti-human neutrophil antigen antibodies, by contrast, are implicated in antibody-mediated transfusion-related acute lung injury (TRALI). This distinction separates recipient sensitisation from donor-antibody-mediated transfusion harm (19).

### 6.2 HLA alloimmunisation and platelet transfusion refractoriness

Platelet transfusion refractoriness should first be confirmed by inadequate post-transfusion increments, ideally using the corrected count increment or percentage platelet recovery after sequential transfusions. Non-immune causes, including sepsis, fever, bleeding, disseminated intravascular coagulation, splenomegaly, medication effects and suboptimal platelet quality, are common and should be excluded before poor increments are attributed to HLA antibodies.

When immune refractoriness is present, anti-HLA class I antibodies are the predominant mechanism because platelets express HLA-A, HLA-B and HLA-C but not usually class II HLA. Antibodies to human platelet antigens (HPA) are less common but clinically important, particularly when HLA-compatible platelets fail. The principal HLA-related clinical questions and practical implications in transfusion medicine are summarised in **Table 3**. Hence, management relies on confirmation of immune refractoriness, definition of antibody specificity and selection of HLA-matched, HLA-compatible, crossmatch-compatible or antibody-specific prediction-selected platelets whenever feasible (23, 24).

**Table 3:** HLA-related questions in transfusion medicine.

Clinical setting	HLA-related mechanism or question	Practical implication
Transfusion exposure	Has the patient been exposed to donor leukocytes or HLA-bearing platelets?	Treat transfusion as a sensitising event, especially in transplant candidates and heavily transfused patients.
Platelet refractoriness	Are anti-HLA class I antibodies present and directed against transfused platelet donor antigens?	Confirm poor increments, exclude non-immune causes, and use HLA-matched, HLA-compatible, crossmatch-compatible or antibody-specific prediction-selected platelets.
Red-cell alloimmunisation	Do recipient HLA class II molecules present peptides derived from transfused red-cell antigens and support CD4 T-cell-dependent alloantibody formation?	HLA-DRB1/DQB1 associations are antigen- and ancestry-dependent. HLA genotyping is investigational and should not currently determine prophylactic red-cell matching or transfusion policy (26–33).
TRALI	Is donor plasma carrying anti-HLA class I, anti-HLA class II or anti-HNA antibodies relevant to the recipient?	Supports transfusion reaction investigation, donor management that is prioritising of plasma product for transfusion derived from male donors and haemovigilance based monitoring strategies.
TA-GVHD	Could viable donor T cells persist because of recipient immunosuppression or donor-recipient HLA sharing?	Use irradiated cellular components for at-risk patients; leukoreduction alone is not adequate prevention.
Neonatal alloimmune neutropenia	Are maternal anti-HNA or anti-HLA antibodies causing fetal/neonatal neutrophil destruction?	Supports specialist granulocyte antibody testing, infection monitoring and neonatal management.
Neonatal alloimmune thrombocytopenia	Are maternal anti-HPA antibodies, with possible anti-HLA contribution, causing fetal platelet destruction?	Supports urgent neonatal platelet support, parental antigen testing and future pregnancy planning.
Transplant candidates	Has transfusion increased cPRA or created DSA risk?	Minimise avoidable transfusion, document exposure history, repeat HLA antibody testing when indicated and coordinate with the transplant laboratory.

**Figure 4:**
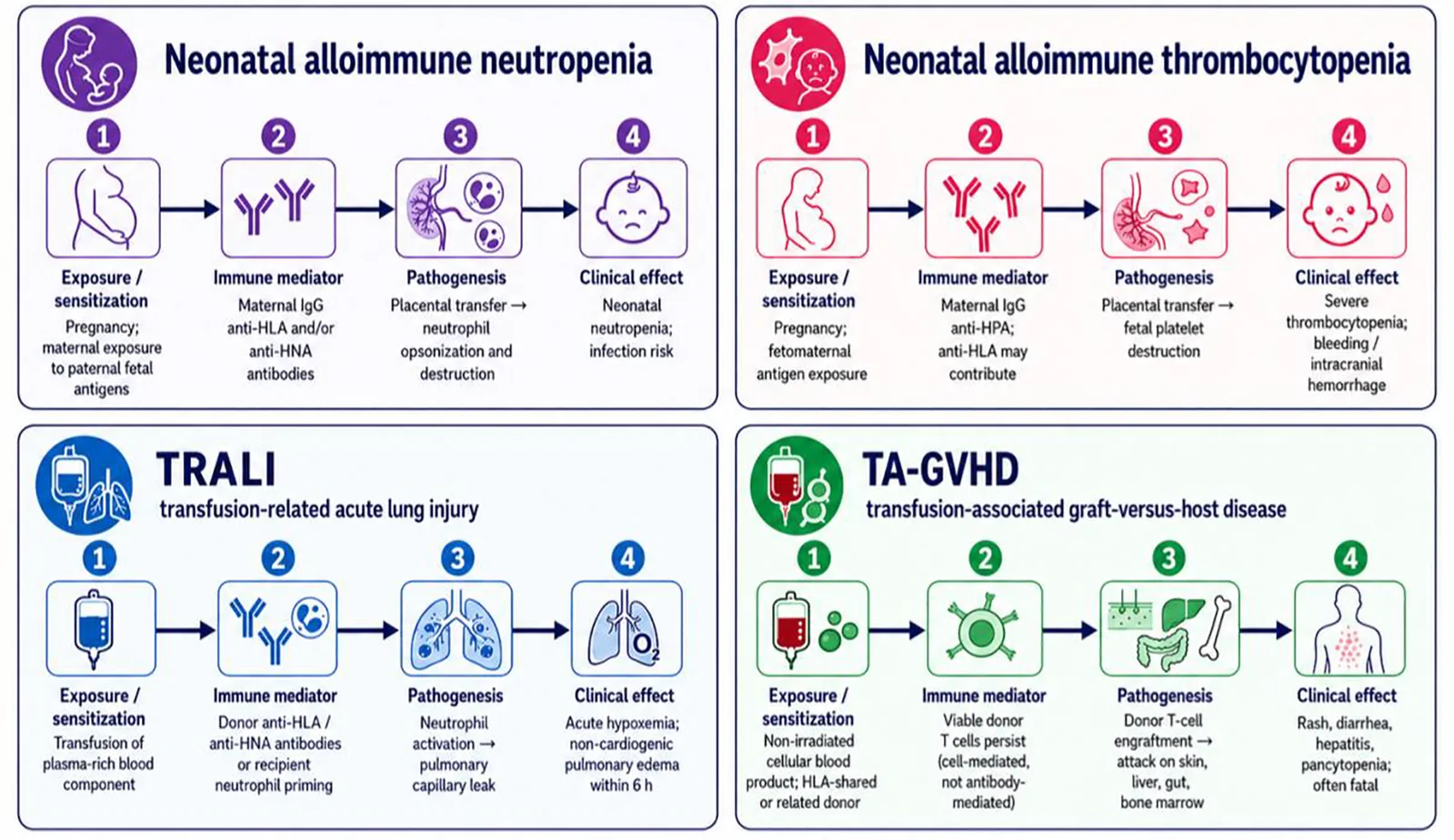
Selected HLA-related transfusion and perinatal alloimmune complications. The image compares exposure or sensitisation, immune mediators, pathogenic mechanisms, and clinical syndromes in neonatal alloimmune neutropenia, fetal and neonatal alloimmune thrombocytopenia, transfusion-related acute lung injury (TRALI), and transfusion-associated graft-versus-host disease (TA-GVHD).

The mechanistic differences between antibody-mediated and cellular HLA-related transfusion complications are summarised in **Figure 4**.

The Trial to Reduce Alloimmunisation to Platelets (TRAP) provides a quantitative anchor: among 530 patients without baseline alloantibodies, alloantibody-associated refractoriness occurred in 13% of controls versus 3-5% in the treated platelet arms, and lymphocytotoxic antibodies developed in 45% versus 17-21%, respectively. These findings established that leukoreduction substantially reduces, but does not eliminate HLA alloimmunisation and alloimmune platelet refractoriness (25). This is supported by a randomised trial and has been incorporated into routine component processing.

By contrast, evidence supporting the optimal platelet-selection strategy after alloimmunisation remains less definitive. Comparative studies of HLA-matched, antibody-compatible and crossmatch-compatible platelet support are derived predominantly from observational cohorts, retrospective analyses and service evaluations, with relatively few randomised comparative trials. Current guidelines recommend individualising platelet-selection strategies based on antibody specificity, transfusion urgency, local donor inventory and laboratory resources rather than favouring a single universally superior approach (23–25).

### 6.3 HLA class II-restricted red-cell alloimmunisation

Red-cell alloimmunisation involves a distinct HLA-dependent pathway from anti-HLA sensitisation caused by residual donor leukocytes. Recipient antigen-presenting cells internalise transfused red-cell proteins, process them into peptides and present selected peptides on HLA class II molecules to CD4 T-cells. Cognate T-cell help can then promote activation, class switching and differentiation of B cells recognising the corresponding red-cell antigen. Human studies identifying HLA class II-restricted helper T-cell epitopes within the RhD and Kell proteins support the biological plausibility of this pathway, although direct evidence remains limited (26, 27).

Consistent with this mechanism, several observational genetic association studies have reported links between recipient HLA class II alleles and susceptibility to alloimmunisation against specific red-cell antigens. HLA-DRB1 and HLA-DQB1 alleles or haplotypes have been associated with alloantibody formation against Fy(a), HLA-DRB1*09:01 with anti-E, HLA-DRB1*03 and HLA-DRB1*04 with anti-D, and HLA-DRB1*15 with the formation of multiple clinically relevant red-cell antibodies (28–31). A systematic review and meta-analysis in sickle cell disease supported a contribution from HLA class II variation, but also identified substantial heterogeneity between studies (32).

However, these findings remain insufficient for routine prediction of red-cell alloimmunisation. The available evidence is derived predominantly from retrospective or case-control studies involving relatively small numbers of alloimmunised patients, frequently focusing on individual blood-group antigens within genetically homogeneous populations. Consequently, reported susceptibility and protective alleles have shown limited reproducibility across different ethnic groups and transfused populations (28–32). Recent computational analysis has identified candidate HLA class II-binding regions within RhD and RhCE proteins, but these predictions require experimental and prospective clinical validation (33). HLA class II genotyping is not currently recommended to guide prophylactic red-cell matching or transfusion policy outside research settings (**Figure 4**).

### 6.4 HLA antibodies and transfusion-related acute lung injury

TRALI illustrates how donor-derived leukocyte antibodies can cause severe inflammatory injury outside transplantation. In the antibody-mediated model, a susceptible recipient receives a plasma-containing component carrying donor anti-HLA class I, anti-HLA class II or anti-human neutrophil antigen (HNA) antibodies. Binding to recipient leukocytes or pulmonary endothelial targets can amplify neutrophil activation, increase endothelial permeability and produce non-cardiogenic pulmonary oedema (34, 35).

The pathogenic antibody is usually donor-derived, and pregnancy-sensitised donors are more likely to carry anti-HLA antibodies. This underpins male-predominant plasma policies, targeted screening or exclusion of higher-risk plasma donors and investigation of implicated donors after suspected reactions. In a prospective active-surveillance study, TRALI incidence fell from 2.57 to 0.81 per 10,000 transfused units after reduced use of plasma from female donors (35). This before-and-after haemovigilance evidence supports donor-risk mitigation at a population level, although it does not provide the causal certainty of a randomised comparison, which would be neither practical nor ethically appropriate in this setting (34, 35).

### 6.5 Transfusion-associated graft-versus-host disease

Transfusion-associated graft-versus-host disease (TA-GVHD) is HLA-related but not antibody-mediated. It occurs when viable donor T-lymphocytes in a cellular blood component persist, engraft, and attack recipient tissues, particularly the skin, gastrointestinal tract, liver, and bone marrow. Fever, rash, diarrhoea, hepatitis and profound pancytopenia are characteristic, and the condition is frequently fatal (36). The HLA basis is distinctive. Donor T-cells are most likely to persist when the recipient is immunodeficient or profoundly immunosuppressed, or when donor and recipient share an HLA haplotype, as may occur after directed donation from a relative or transfusion from an HLA-homozygous donor.

Prevention requires irradiation of at-risk cellular blood components to prevent donor lymphocyte proliferation; leukoreduction alone is insufficient. TA-GVHD therefore represents the cellular counterpart to antibody-mediated HLA complications such as platelet refractoriness and TRALI (36). Because TA-GVHD is rare, unpredictable and associated with extremely high mortality, randomised prophylactic trials are neither feasible nor ethical. Hence, recommendations are supported by pathophysiology, case reports, systematic reviews and haemovigilance experience, rather than randomised trials. The absence of trial data should not be interpreted as uncertainty about prophylaxis in recognised risk groups (36).

### 6.6 HLA, granulocyte antibodies and neonatal alloimmune cytopenias

Neonatal alloimmune neutropenia usually results from maternal immunoglobulin G (IgG) antibodies directed against paternally inherited fetal human neutrophil antigens (HNA). These antibodies cross the placenta and destroy fetal or neonatal neutrophils. HNA antibodies are the principal mechanism, although anti-HLA antibodies may coexist or contribute in selected cases. The phenotype ranges from incidental neutropenia to serious infection risk, and evaluation may require maternal antibody testing, specialist granulocyte immunology and exclusion of maternal autoimmune or sepsis-related neonatal neutropenia (37).

Fetal and neonatal alloimmune thrombocytopenia is usually mediated by maternal IgG antibodies against paternally inherited HPA on fetal platelets, particularly HPA-1a in many populations. Antibody binding can cause severe thrombocytopenia, bleeding and intracranial haemorrhage. Anti-HLA class I antibodies may be detected and can complicate interpretation, but they are not the dominant mechanism in typical disease. Severely affected neonates may require urgent antigen-compatible platelet support, and subsequent pregnancies require specialist planning (38, 39). The evidence base consists mainly of specialist cohorts and case series, and the clinical contribution of anti-HLA antibodies is less consistent than that of HPA or HNA antibodies (37–39).

### 6.7 Practical interpretation

HLA-related transfusion complications arise through distinct immune mechanisms. Platelet refractoriness and TRALI are predominantly antibody-mediated; TA-GVHD is mediated by viable donor T cells; neonatal cytopenias are usually caused by maternal alloantibodies; and recipient HLA class II molecules may influence red-cell alloantibody formation. Diagnostic and preventive strategies should therefore be directed towards the relevant mechanism.

## 7 HLA and disease association

### 7.1 Autoimmunity: from association to clinical use

The HLA region contains some of the strongest genetic associations with autoimmunity, but their clinical meaning differs substantially by disease. HLA variation can alter the self-peptide repertoire, thymic selection and peripheral T-cell responses, thereby influencing loss of tolerance and autoantibody formation. Most associations modify susceptibility or phenotype and are not sufficiently specific or penetrant to establish a diagnosis on their own. Selected HLA-disease and pharmacogenomic associations, with their evidence base are summarised in **Table 4**.

**Table 4:** Evidence appraisal of selected HLA-disease and pharmacogenomic associations.

Clinical area	Representative HLA findings	Evidence base and validation	Current clinical use, limitations and unresolved questions
Autoimmunity	Type 1 diabetes: HLA-DR3-DQ2 and HLA-DR4-DQ8 risk; HLA-DQB1*06:02 protection. Systemic lupus erythematosus: HLA-DRB1*03:01/HLA-DRB1*15:01 and peptide-binding groove residues. Coeliac disease: HLA-DQ2/DQ8. Spondyloarthritis: HLA-B*27.	Associations are supported by large genetic studies and, for several diseases, mechanistic work. Effect sizes and allele frequencies vary by ancestry, phenotype definition and linkage disequilibrium (4, 40–42).	HLA testing can modify diagnostic probability or exclude selected diagnoses, but is rarely diagnostic alone. Type 1 diabetes and lupus associations are not routine stand-alone tests; ancestry calibration and phenotype-specific validation remain important.
Infection	HLA-B*57/B*27 and HIV control; HLA-restricted responses in hepatitis C virus infection; HLA-B*53 and protection from severe malaria.	Evidence is derived mainly from observational cohorts, population studies and mechanistic analyses of peptide presentation and viral escape (43–45).	These findings illuminate host-pathogen biology but generally do not guide routine diagnosis or treatment. Transferability is limited by pathogen, population and HLA-frequency differences.
Pharmacogenomics	HLA-B*57:01-abacavir; HLA-B*15:02/HLA-A*31:01-carbamazepine; HLA-B*58:01-allopurinol; HLA-A*32:01-vancomycin DRESS; HLA-B*13:01-dapsone/co-trimoxazole severe cutaneous adverse reactions.	Abacavir and carbamazepine are supported by prospective screening studies and guideline implementation. Evidence for vancomycin and some HLA-B*13:01 applications is based mainly on case-control or population-specific cohorts (46–53).	Testing is established only where clinical utility and prescribing pathways are defined. Population prevalence, positive predictive value, access and availability of alternative drugs determine implementation.
HLA evolutionary divergence	Higher class I HED in cancer immunotherapy; donor class I HED in liver rejection; HED in HSCT; HLA-DP diversity in vaccine response.	Evidence is predominantly retrospective and observational, with differing loci, calculations, endpoints and treatment platforms (12–15).	No validated universal threshold or decision rule exists. HED remains investigational and requires prospective, disease- and organ-specific validation.

Type 1 diabetes was among the earliest diseases linked to HLA. Susceptibility is dominated by class II haplotypes, particularly HLA-DR3-DQ2 and HLA-DR4-DQ8, whereas HLA-DQB1*06:02 is strongly protective in many populations. These effects reflect the peptide-binding properties of HLA-DQ and HLA-DR molecules and their influence on islet-antigen presentation and thymic selection. HLA contributes substantially to genetic risk scores used in research, screening and classification of atypical diabetes, but predictive performance differs across ancestral populations and complements rather than replaces clinical, serological and metabolic assessment (40).

Systemic lupus erythematosus (SLE) provides a more polygenic and heterogeneous example. HLA-DRB1*03:01 and HLA-DRB1*15:01 are recurrent susceptibility signals and are associated with particular autoantibody profiles, while fine-mapping has localised much of the MHC association to amino-acid positions within the HLA-DRβ1 peptide-binding groove (41, 42). In coeliac disease, absence of HLA-DQ2/DQ8 can help exclude disease in selected settings; HLA-B*27 in spondyloarthritis and HLA-DRB1 shared-epitope alleles in rheumatoid arthritis mainly modify diagnostic probability or phenotype (4). These associations are well replicated, but their clinical utility is uneven, and their effect estimates vary with ancestry. Consequently, HLA typing is best regarded as an adjunctive biomarker for diagnosis, classification or prognostication rather than a stand-alone diagnostic test in most autoimmune diseases (4, 41, 42).

### 7.2 HLA in infection

In infection, HLA class I molecules shape cytotoxic control and viral escape. Most evidence derives from observational association studies and genome-wide analyses, and many reported associations vary according to pathogen diversity, host ancestry and local selective pressures. HLA-B*57 and HLA-B*27 have been associated with slower human immunodeficiency virus (HIV) progression; HLA-restricted T-cell responses influence hepatitis C virus control and escape; and HLA-B*53 has been associated with protection from severe malaria in West Africa (43–45). These examples illustrate how the same principles of peptide binding and immune selection can produce protective, detrimental or population-specific effects. Yet they may have limited routine clinical utility outside research settings.

### 7.3 Pharmacogenomics and precision prescribing

HLA pharmacogenomics is among the clearest examples of precision immunology in clinical practice. In the PREDICT-1 trial, prospective HLA-B*57:01 screening eliminated immunologically confirmed abacavir hypersensitivity in the screened group (0% versus 2.7% in controls) and reduced clinically suspected hypersensitivity from 7.8% to 3.4% (46). In a prospective Taiwanese carbamazepine-screening programme, 4,877 candidates were tested before treatment; 7.7% were HLA-B*15:02-positive and avoided carbamazepine, and no cases of Stevens-Johnson syndrome or toxic epidermal necrolysis occurred, compared with approximately 10 expected from historical incidence (47). Established guidance supports HLA-B*57:01 testing for abacavir, HLA-B*15:02 and HLA-A*31:01 testing for carbamazepine or oxcarbazepine, and HLA-B*58:01 testing for allopurinol risk management (48–50). HLA-A*32:01 is strongly associated with vancomycin-induced drug reaction with eosinophilia and systemic symptoms (DRESS), but currently has greater value for culprit-drug attribution than for universal pre-prescribing screening (51). HLA-B*13:01 is associated with dapsone hypersensitivity and has been linked to severe cutaneous adverse reactions to co-trimoxazole in selected populations (52, 53). Clinical translation depends on more than association strength: the test must be reproducible, the risk estimate population-specific, an alternative treatment available and patient outcomes improved by testing. The evidentiary hierarchy is not uniform. Abacavir and carbamazepine screening are supported by prospective implementation studies and guideline adoption, while allopurinol testing is established in defined high-risk populations. HLA-A*32:01 and HLA-B*13:01 are supported mainly by case-control or population-specific studies; their value outside the studied populations and clinical contexts remains less certain (46–53).

## 8 HLA in cancer and immunotherapy

HLA has become an increasingly important focus in cancer immunology because cytotoxic T-cell recognition requires tumour antigens to be processed, presented and retained on HLA molecules at the tumour-cell surface. Tumours may evade this surveillance through loss of HLA class I expression, disruption of β2-microglobulin, defects in antigen processing, HLA loss of heterozygosity or suppression of interferon-responsive presentation pathways (10). Tumour mutational burden alone is therefore an incomplete measure of immunogenicity: a candidate neoantigen must bind the patient’s HLA molecules and remain displayed by the malignant clone. HLA evolutionary divergence adds a germline component, with more divergent class I alleles associated with broader peptide presentation and improved checkpoint-inhibitor outcomes in some cohorts, although the effect remains tumour- and treatment-dependent (12). Loss of classical HLA and defects in antigen processing are supported by extensive mechanistic and clinical evidence. By contrast, HED as a predictive biomarker is based mainly on retrospective cohorts, and prospective evaluation of a clinically usable threshold is lacking (10, 12).

Unlike classical HLA molecules, which present peptide antigens, non-classical HLA molecules have predominantly regulatory functions. HLA-G can inhibit T-cells, natural killer (NK) cells and antigen-presenting cells through immunoglobulin-like transcript receptors, whereas HLA-E engages CD94/NKG2A on NK cells and cytotoxic T-cell subsets, thereby reducing antitumour effector activity (11, 54, 55). The HLA-E–NKG2A pathway is therefore being investigated as a therapeutic target, and anti-NKG2A blockade has been shown to enhance NK-cell and T-cell antitumour responses (55, 56). Single-cell RNA-sequencing studies in pancreatic ductal adenocarcinoma and biliary tract cancer have identified HLA-E expression within the tumour microenvironment and suggest a candidate HLA-E–NKG2A regulatory axis in γδ T-cell- and NK-cell-rich immune niches (57, 58). Although γδ T-cells are not classically HLA-restricted in the same manner as αβ T cells, NKG2A-expressing subsets may still be inhibited through HLA-E engagement (55, 57, 58). These observations remain hypothesis-generating, as they derive from discovery datasets without independent clinical validation, and they do not currently support routine measurement of tumour HLA-E or NKG2A for treatment selection.

## 9 HLA in solid organ transplantation

Solid organ transplantation is the clinical setting in which HLA information most directly informs donor selection, immunological risk stratification and post-transplant surveillance. Donor-recipient HLA disparity can initiate direct, indirect and semi-direct allorecognition, leading to cellular rejection, donor-specific antibody formation, antibody-mediated rejection (ABMR) and chronic graft injury (59, 60). The evidentiary hierarchy within transplantation is nevertheless uneven. Conventional antibody screening, physical or virtual crossmatching and unacceptable-antigen assignment are established components of care, whereas molecular-mismatch scores, donor-derived cell-free DNA and genetically predicted innate allorecognition remain adjunctive or investigational. These distinctions are examined in the following sections.

Kidney transplantation provides the clearest evidence base for clinically meaningful HLA assessment. HLA-DR and HLA-DQ mismatches are associated with *de novo* DSA formation, ABMR and long-term graft loss. Contemporary practice integrates antibody screening, unacceptable-antigen listing, cPRA, virtual crossmatching and peri-transplant physical crossmatch results, while recognising that ischaemia-reperfusion injury, adherence, immunosuppression, infection, recurrent disease and non-HLA antibodies also modify risk (61–63). These associations arise largely from observational cohorts, but their reproducibility across centres and incorporation into allocation and monitoring pathways support clinical utility, particularly in kidney transplantation (61–63).

Sensitisation history links transfusion medicine directly with transplant immunology because previous immune exposures shape both the likelihood and strength of anti-HLA responses. Prior transplantation, pregnancy and transfusion have different effects on antibody breadth, panel-reactive antibody profiles and access to transplantation (64–66). In a 1,066-case renal transplant cohort, positivity across Luminex panel-reactive antibody (L-PRA), FC-XM and CDC-XM was higher after retransplantation (73.91%, 100% and 56.52%), pregnancy (57.46%, 70.14% and 18.85%) or transfusion (27.33%, 35.55% and 7.33%) than among candidates without a recognised sensitising event (6.17%, 13.58% and 1.09%). Prior transplantation produced the strongest alloimmune profile (64). These data support detailed documentation of exposure history and interpretation of antibody results alongside specificity, strength, dilution behaviour and assay context (64–66). No single assay is sufficient: CDC-XM is specific but insensitive, FC-XM is more sensitive but may be non-specific, and L-SAB defines specificity but remains vulnerable to interference, epitope sharing and semi-quantitative MFI interpretation (17–22).

Molecular mismatch analysis refines antigen-level matching but remains methodologically heterogeneous. Eplets describe polymorphic configurations on the HLA surface; predicted indirectly recognisable HLA epitopes presented by HLA class II (PIRCHE-II) estimates donor HLA-derived peptides that may be presented by recipient class II molecules; and the HLA epitope mismatch algorithm (HLA-EMMA) and Snow/Snowflake approaches quantify solvent-accessible amino-acid or structural disparity. Higher HLA-DQ and class II molecular mismatch loads are associated with *de novo* DSA and antibody-mediated injury, but thresholds, algorithm versions, typing resolution and organ-specific performance remain unsettled (67–73). A recent implementation review identified potential roles in deceased-donor allocation, living-donor selection, access for highly sensitised candidates and post-transplant risk stratification but concluded that prospective evidence remains insufficient for routine allocation or immunosuppression tailoring (74). The 2025 Sensitisation in Transplant: Assessment of Risk (STAR) report reached a similar conclusion: current scores quantify disparity more readily than biological immunogenicity and should not be used in isolation for individual treatment or allocation decisions (75).

Population structure is central to implementation. HLA frequencies, linkage disequilibrium and donor availability differ by ancestry, and allocation systems that reward close matching can disadvantage patients from underrepresented groups. Many molecular mismatch algorithms have been developed and validated predominantly in European ancestry cohorts. Molecular tools may improve biological resolution, but eplet, amino-acid, Snow/Snowflake, and PIRCHE-II methods can classify the same donor-recipient pair differently. Their use in allocation should therefore be prospectively evaluated for both clinical benefit and distributional effects across populations (73–75).

Recent consensus also places HLA results within a broader biomarker framework. Circulating HLA antibodies remain the established non-invasive marker of alloimmune response, but MFI should be interpreted with physical crossmatch results, serum dilution, reactivity patterns, prior exposure and graft phenotype. Donor-derived cell-free DNA (dd-cfDNA) may indicate allograft injury, but it is not specific for rejection and remains assay- and context-dependent; it should complement rather than replace DSA assessment, histology and clinical evaluation (75).

The STAR 2025 report also highlighted innate allorecognition as a potential contributor to graft injury. Recipient NK cells may become activated when inhibitory KIRs lack cognate donor HLA class I ligands, a “missing-self” configuration that may contribute to microvascular inflammation with or without DSA. Genetic prediction requires donor and recipient HLA class I typing together with recipient KIR genotyping, and current evidence does not support routine clinical testing (75).

### 9.1 Organ-specific implications of HLA mismatch

The clinical importance of HLA disparity varies by organ type, donor source and urgency. Evidence is strongest in kidney transplantation, whereas studies in liver, pancreas, heart, lung and corneal transplantation are predominantly observational and less consistent. **Table 5** compares the evidence base, current clinical use, guideline support and remaining uncertainties across organs.

**Table 5:** Critical appraisal of organ-specific HLA mismatch and antibody evidence.

Organ/source	Principal HLA-related concern	Evidence base and validation	Current clinical use	Guideline support	Key limitations and unresolved questions
Kidney	HLA-DR/DQ mismatch, preformed and *de novo* DSA predict ABMR, chronic graft injury and graft loss.	Strongest evidence base. Large multicentre longitudinal cohorts, registry studies and independent validation consistently demonstrate associations. Molecular mismatch (particularly HLA-DQ eplets) provides additional prognostic information but is supported mainly by observational studies.	Routine HLA typing, antibody screening, unacceptable-antigen assignment, cPRA, virtual/physical crossmatch and post-transplant DSA surveillance are standard practice. Molecular mismatch is used selectively for adjunctive risk stratification.	Established international guideline support for conventional HLA assessment; molecular mismatch not yet routinely recommended.	Optimal molecular thresholds, algorithm harmonisation, timing of surveillance and integration into immunosuppression decisions remain uncertain.
Liver	Persistent class II DSA may contribute to chronic rejection and graft injury despite relative tolerance to HLA mismatch.	Moderate evidence is based mainly on small prospective or retrospective cohorts. In one 90-recipient study, 22.2% had preformed donor-specific antibodies; persistent high antibody levels were associated with diffuse C4d deposition. Donor class I HED has been associated with rejection in a retrospective cohort (13, 76).	HLA matching is not routinely used for allocation. DSA testing is performed selectively in clinically indicated settings.	Limited guideline support for routine HLA-based monitoring.	The incremental value of routine donor-specific antibody surveillance and HED requires independent prospective evaluation.
Pancreas	DSA associated with rejection and inferior graft survival, particularly in simultaneous pancreas-kidney transplantation and isolated pancreas transplantation.	Evidence is limited and largely single-centre. A cohort of 167 deceased-donor recipients reported more rejection and lower graft survival in donor-specific antibody-positive patients (77).	Antibody monitoring is performed in many centres, but HLA matching is constrained by donor availability and urgency.	Limited evidence-based guidance.	Universal surveillance strategies, intervention thresholds and molecular mismatch utility remain undefined.
Heart	Preformed and *de novo* DSA contribute to ABMR, cardiac allograft vasculopathy and graft dysfunction.	Guideline recommendations are supported mainly by observational cohorts and consensus interpretation rather than randomised evidence. Associations with antibody-mediated rejection and adverse long-term outcomes have been reproduced (78, 79).	Unacceptable-antigen listing, virtual/rapid crossmatch and DSA surveillance are widely implemented.	Supported by consensus guidelines, although based largely on observational evidence.	Clinical significance of subclinical DSA and assay thresholds remain uncertain.
Lung	HLA-DQ DSA and antibody-mediated rejection are strongly associated with chronic lung allograft dysfunction (CLAD) and graft failure.	Evidence is observational and heterogeneous in assay methods and outcome definitions. In one cohort, 17% developed donor-specific antibodies, which were associated with worse chronic lung allograft dysfunction-free survival (80).	Antibody profiling and surveillance are widely used.	Moderate guideline support, but practice varies.	Optimal surveillance frequency, treatment thresholds and benefit of treating asymptomatic antibodies are uncertain.
Living vs deceased donor	The same immunological profile carries different practical implications according to donor source, urgency and available alternatives.	This distinction reflects allocation logistics and accumulated clinical evidence rather than a single comparative study (78, 81).	Living donation enables planned HLA assessment, paired exchange and desensitisation; deceased donation requires rapid, context-dependent decisions while balancing immunological risk.	Integrated into allocation policies rather than disease-specific guidelines.	Balancing immunological optimisation, equity, organ utilisation and waiting-list mortality remains a continuing challenge.
Cornea	HLA matching may matter in vascularised, inflamed or repeat graft beds, while primary avascular grafts are relatively immune-privileged.	Evidence is limited, includes older studies and is concentrated in selected high-risk recipients. One study reported higher two-year rejection-free survival with better HLA-A/B matching (82).	Routine matching is unnecessary for low-risk grafts; selective matching may be considered for high-risk recipients.	Selective recommendations for high-risk transplantation only.	Contemporary validation is needed for high-risk populations and modern immunosuppressive practice.

## 10 HLA in haematopoietic stem cell transplantation

Haematopoietic stem cell transplantation (HSCT) differs from solid organ transplantation because the graft transfers a functioning donor immune system. HLA disparity can cause graft failure, graft-versus-host disease (GVHD) and mortality, but it may also contribute to graft-versus-leukaemia effects. Donor selection must therefore balance histocompatibility with disease urgency, donor availability, graft source, conditioning intensity and GVHD prophylaxis (83, 84). Randomised donor-selection trials are not feasible, but the relationship between high-resolution matching and outcome has been reproduced in large registry and multicentre cohorts and is incorporated into donor-selection guidance (83–86).

The practical impact of HLA matching also varies across populations because HLA allele frequencies and unrelated donor availability differ substantially between ancestral groups, influencing the likelihood of identifying optimally matched donors. For unrelated donor transplantation, high-resolution matching at HLA-A, HLA-B, HLA-C and HLA-DRB1 remains central, with additional consideration of HLA-DQB1 and HLA-DPB1. Among class II loci, HLA-DPB1 is distinctive because selected mismatches are considered permissive according to T-cell epitope models. Current European Society for Blood and Marrow Transplantation (EBMT) recommendations therefore integrate HLA-DPB1 permissiveness with donor age, graft source, donor availability and GVHD prophylaxis rather than considering HLA compatibility in isolation (85, 86).

Donor-specific anti-HLA antibodies are a major risk factor for graft failure in mismatched unrelated, cord blood and haploidentical HSCT. Screening is particularly important when donor-recipient mismatch exists. If no alternative donor is available, desensitisation may be attempted, but evidence remains heterogeneous, and the urgency of the underlying disease often determines practice (87–89). The association between DSA and graft failure has been reproduced in several observational cohorts. In contrast, desensitisation strategies are supported mainly by heterogeneous single-centre protocols without a common universally accepted endpoint or comparative standard (87–89).

Haploidentical transplantation, particularly with post-transplant cyclophosphamide (PTCy), has expanded donor availability without removing the influence of HLA. Donor selection increasingly considers age, relationship, sex, cytomegalovirus serostatus, ABO blood group compatibility, DSA, KIR-ligand interactions, HLA-DPB1 permissiveness and disease-specific priorities (86, 90). Emerging variables include HED, which may influence GVHD, relapse and graft-versus-leukaemia balance (14), and HLA-B leader dimorphism, which links class I leader peptides with HLA-E-mediated NK-cell regulation (91). HED and HLA-B leader dimorphism remain emerging modifiers and should not displace established donor factors until prospectively validated within specific graft-source and prophylaxis platforms (14, 91). Accordingly, high-resolution HLA matching, DSA assessment and HLA-DPB1 permissive mismatch represent established components of contemporary donor selection, whereas HED and HLA-B leader dimorphism remain investigational modifiers pending further clinical evaluation before translation to practice.

## 11 HLA, pregnancy and physiological tolerance

Pregnancy provides a physiological model of semi-allogeneic tolerance in which non-classical HLA molecules help regulate the maternal immune response to the fetus. Extravillous trophoblasts have a distinctive HLA profile: they do not express classical HLA-A or HLA-B, but retain HLA-C and express HLA-E, HLA-F and HLA-G. This pattern limits conventional T-cell recognition while permitting controlled interactions with decidual natural killer cells and other maternal immune populations. HLA-G is particularly important in this setting because it can suppress cytotoxic responses, promote tolerogenic antigen-presenting-cell phenotypes and support placental development (11).

Maternal killer cell immunoglobulin-like receptor (KIR) and fetal HLA-C combinations influence decidual natural killer-cell activation, trophoblast invasion and vascular remodelling. Associations with recurrent pregnancy loss, pre-eclampsia and fetal growth restriction have been reported, but the evidence is observational and varies across populations; routine reproductive testing is therefore not established (92). Pregnancy is also an important source of HLA sensitisation, as exposure to paternally derived fetal HLA can generate maternal anti-HLA antibodies that may later complicate transfusion support or transplant compatibility (64, 65, 92). These mechanisms also inform current research into HLA-mediated immune suppression in cancer and tolerance in transplantation.

## 12 Precision medicine and clinical translation

Clinical translation of an HLA biomarker should proceed from discovery through analytical validity and clinical validity to demonstrable clinical utility. Pharmacogenomic tests such as HLA-B*57:01 have traversed much of this pathway because they are technically reproducible, alter prescribing and prevent a defined adverse outcome. By contrast, HED, molecular mismatch scores, HLA-E/HLA-G expression, and many functional antibody assays remain at earlier stages. Association with outcome is not sufficient unless a test is reproducible across laboratories, changes a clinical decision, improves patient outcomes and is feasible within the relevant health system (74, 75). Clinical readiness should therefore be judged by the weakest link in this pathway rather than by association strength alone.

Precision HLA medicine will probably rely on integrated models that combine several sources of information. Relevant data may include donor and recipient HLA sequences, HED, eplet or solvent-accessible amino-acid mismatch, PIRCHE-II, antibody specificity and titre, complement activation, B-cell memory, T-cell help, innate immune activation, organ-injury markers and exposure history. Such models require prospective calibration within defined organs, diseases and treatment platforms (12–15, 67–75). Most of these integrated approaches remain investigational and require sufficient evidence before routine implementation.

Functional interpretation of HLA antibodies remains a particular need. Future assays should distinguish immunological relevance rather than antibody presence alone. L-SAB assays define specificity, but similar MFI values may have different clinical consequences. Serum dilution, complement binding, immunoglobulin subclass, fragment crystallisable (Fc) glycosylation, epitope density and endothelial activation may help distinguish clinically injurious from permissive antibodies. The STAR 2025 report emphasised that MFI is semi-quantitative, should not be summed across antibodies, and should be interpreted with crossmatch results, reactivity patterns, prior exposure and clinical phenotype (17–19, 60, 75).

Population diversity is a scientific and implementation requirement. Allele and haplotype frequencies, linkage disequilibrium and donor availability differ across populations, while many disease cohorts, HLA reference panels and donor registries remain unrepresentative. Risk scores, molecular-mismatch thresholds and imputation models should therefore be validated across ancestries, and allocation systems should be monitored for differential effects on access and outcomes (5, 6, 73–75).

In oncology, therapeutic development may exploit HLA-restricted neoantigens, restoration of antigen presentation and HLA-E/HLA-G checkpoint pathways (10–12, 54–56). Pharmacogenomics testing may expand towards pre-emptive panels, but only where prospective evidence shows that test-guided prescribing improves outcomes (46–53). In transplantation, tolerance-inducing strategies remain investigational and require evaluation against clinically meaningful endpoints (59, 75).

A complementary way to interpret the evidence is to distinguish clinically actionable HLA information from associations that remain primarily mechanistic or investigational. **Table 6** summarises settings in which HLA results currently change prescribing, transfusion support, donor selection, transplant compatibility assessment, surveillance or therapeutic planning. The evidence category reflects study design, independent validation, assay standardisation and evidence that the result changes management or outcomes.

**Table 6:** Evidence hierarchy and current clinical use of HLA information.

Clinical setting	HLA information	Evidence level, validation and guideline status	Current clinical use and unresolved questions
Abacavir prescribing	HLA-B*57:01	Established: prospective randomised screening evidence, independent replication and guideline implementation (46, 48).	Do not prescribe abacavir to positive patients. Residual issues relate mainly to equitable access and testing logistics.
Carbamazepine/oxcarbazepine	HLA-B*15:02 and HLA-A*31:01	Established in genetically at-risk populations: prospective screening evidence and guideline support; clinical utility depends on allele prevalence (47, 49).	Use genotype-guided prescribing where population risk justifies testing. Predictive value is lower in populations in which the risk alleles are uncommon.
Red-cell alloimmunisation risk prediction	Recipient HLA-DRB1/DQB1 alleles and predicted presentation of red-cell antigen-derived peptides	Investigational: mechanistic HLA class II-restricted T-cell studies, antigen-specific case-control associations, a systematic review/meta-analysis in sickle cell disease and recent in silico work; findings vary by antigen and ancestry (26–33).	HLA genotyping does not currently guide prophylactic red-cell matching. Larger multi-ancestry prospective studies and experimental validation of predicted epitopes are required.
Allopurinol	HLA-B*58:01	Established for selected high-risk populations: strong replicated association and guideline support (50).	Screen populations in whom prevalence and clinical benefit justify testing; select an alternative for positive patients when suitable.
Platelet transfusion refractoriness	Anti-HLA class I antibody specificity	Established transfusion-support practice: strong mechanistic evidence and extensive clinical experience; comparative trials of selection strategies are limited (23–25).	Use HLA-matched, HLA-compatible, crossmatch-compatible or antibody-specific prediction-selected platelets according to antibody profile and donor inventory.
TRALI prevention and investigation	Donor anti-HLA/anti-HNA antibodies	Established haemovigilance practice: supported by active-surveillance and policy studies rather than randomised trials (34, 35).	Guide donor investigation and plasma-risk mitigation. Individual antibody detection does not by itself establish causality.
Kidney transplantation	Preformed donor-specific antibodies, unacceptable antigens, cPRA and crossmatch results	Established: replicated cohort evidence, assay integration and consensus-based clinical pathways (59–64).	Exclude donors expressing unacceptable antigens, consider desensitisation when appropriate and tailor surveillance. Centre-specific assay interpretation remains necessary.
Molecular mismatch in kidney transplantation	HLA-DQ eplet load, PIRCHE-II and HLA-EMMA/Snow-related approaches	Evolving: mainly retrospective observational cohorts; algorithms, versions and thresholds are not standardised, and prospective clinical utility remains unproven (67–75).	May support counselling and surveillance, but should not independently determine allocation or immunosuppression outside validated protocols.
Unrelated donor HSCT	High-resolution HLA-A, -B, -C, -DRB1 and selected class II matching	Established: large registry and multicentre observational evidence with guideline integration (83–86).	Prioritise compatible donors while balancing donor age, graft source, urgency, disease risk and graft-versus-host disease prophylaxis.
Mismatched, cord blood or haploidentical HSCT	Donor-specific anti-HLA antibodies	Clinically important risk marker: associations with graft failure have been reproduced in observational cohorts; desensitisation evidence is heterogeneous (87–89).	Select another donor where possible. When no alternative exists, desensitisation should be individualised and supported by serial antibody assessment.
HLA-DPB1 in HSCT	Permissive versus non-permissive HLA-DPB1 mismatch	Established donor-selection modifier in selected unrelated-donor settings: retrospective multicentre validation and guideline integration (85, 86, 90).	Use with donor age, availability, graft source and prophylaxis platform; its effect may differ with post-transplant cyclophosphamide.
Cancer and pregnancy tolerance	HLA-G/HLA-E expression and regulatory pathways	Exploratory: mechanistic, translational and early therapeutic evidence without validated routine biomarker thresholds (11, 54, 55).	Useful for biological interpretation and trial design, but these markers do not currently guide routine clinical selection in most settings.
Vancomycin DRESS	HLA-A*32:01	Emerging: case-control evidence supports culprit-drug attribution, but universal pre-prescribing screening has not been validated (51).	May support attribution in complex DRESS cases and future drug selection; prospective implementation studies are needed.
Dapsone/co-trimoxazole severe cutaneous adverse reactions	HLA-B*13:01	Population-dependent: replicated in selected populations, with limited evidence for universal generalisability or screening (52, 53).	Consider genotype-guided alternatives where allele prevalence, drug use and local implementation justify testing.
HED-based risk stratification	Class I/II HLA evolutionary divergence	Exploratory: retrospective associations using heterogeneous calculations and endpoints, without a validated threshold or prospective utility study (12–15).	May inform research models in immunotherapy, liver transplantation and HSCT; it should not yet direct routine treatment or allocation.
Type 1 diabetes risk assessment	HLA-DR/DQ haplotypes within validated genetic risk scores	Investigational: genetic risk-score studies support research screening or classification, but performance varies by ancestry and clinical context (40).	May assist research or atypical-disease classification; it is not diagnostic alone and requires ancestry-calibrated models.

## 13 Limitations of the current evidence base

Several limitations recur across HLA research. First, extensive linkage disequilibrium across the MHC can obscure the causal allele, amino acid residue, regulatory variant, or neighbouring gene. Second, HLA allele and haplotype distributions differ between populations, yet many disease cohorts, reference panels and donor registries remain weighted towards European ancestry; inadequate control of population structure can produce biased or poorly transferable estimates. Third, typing resolution and imputation accuracy vary across studies and may alter HED or molecular-mismatch calculations. Fourth, clinical phenotypes such as rejection, drug hypersensitivity, autoimmune disease and platelet refractoriness are heterogeneous and are not always adjudicated consistently. Finally, most molecular-mismatch and HED studies are observational, and prospective intervention studies are needed before these measures can guide allocation or treatment. These limitations may explain why apparently robust HLA associations are not always reproducible across diseases, populations or clinical settings.

In transplant immunology, CDC-XM, FC-XM and L-SAB measure related but distinct phenomena. Denatured antigens, complement interference, prozone, bead saturation, epitope sharing, reagent lots and platform differences can all affect interpretation. Emerging biomarkers and algorithms add information but do not remove the necessity and importance of clinical context. Multidisciplinary interpretation by histocompatibility scientists, transfusion specialists, physicians and transplant teams remains essential.

## 14 Conclusions

HLA biology has evolved from a fundamental immunogenetic concept into an important component of precision medicine across transplantation, transfusion medicine, pharmacogenomics, oncology and autoimmune disease. HLA biology aids clinical decisions across medicine, from drug selection and platelet support to donor assessment, autoimmune disease interpretation and cancer immunology. These applications are related but not interchangeable: allele-level disease risk, anti-HLA antibodies, molecular disparity and non-classical regulatory pathways answer different clinical questions.

Clinical implementation should remain proportionate to the quality and consistency of supporting evidence. Established pharmacogenomic tests and transplant compatibility methods already alter care, whereas HED, molecular-mismatch algorithms, HLA-E/HLA-G biomarkers and innate allorecognition remain investigational. Progress will depend on standardised assays, prospective evaluation of clinical utility, population-representative data and equitable access to donors, testing and targeted therapies. This hierarchy should also determine the next step: established tests should guide care; evolving biomarkers should be evaluated in prospective implementation studies; and exploratory mechanisms cannot define immediate clinical decisions, but they should define research priorities.
